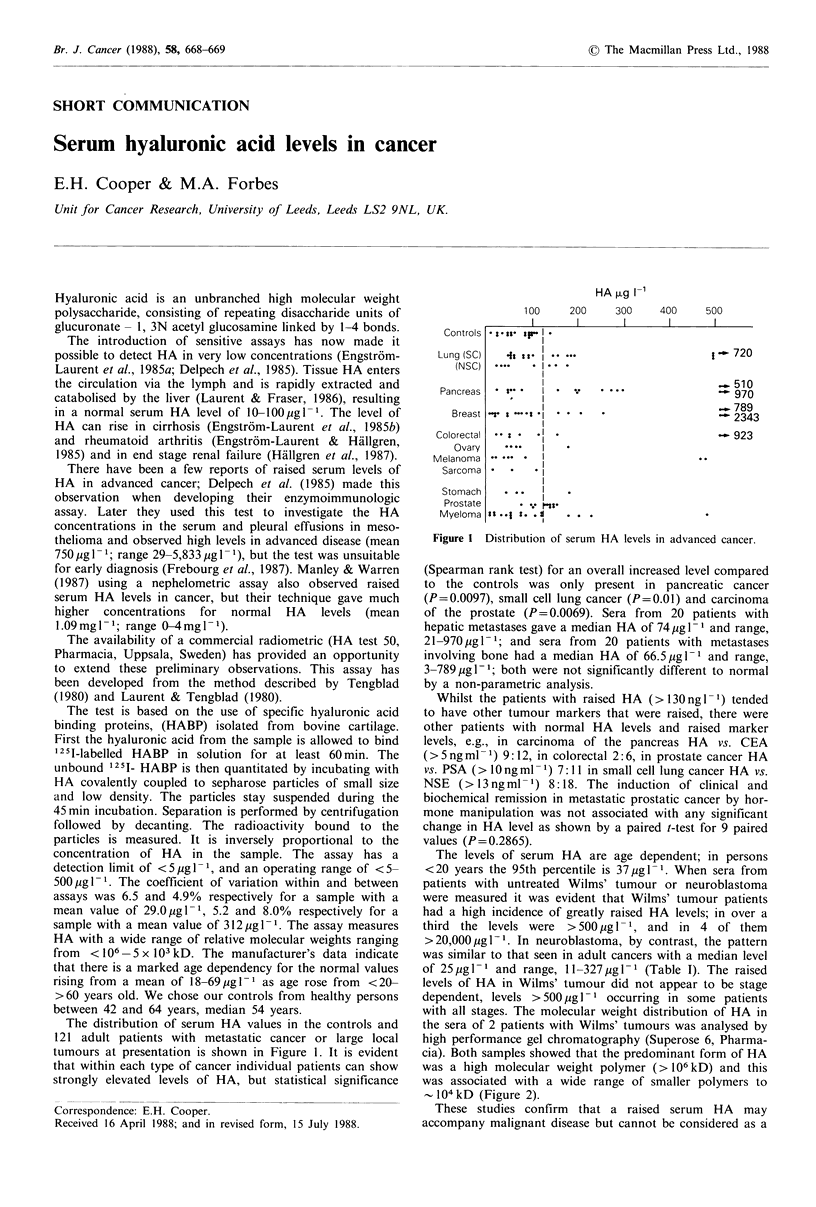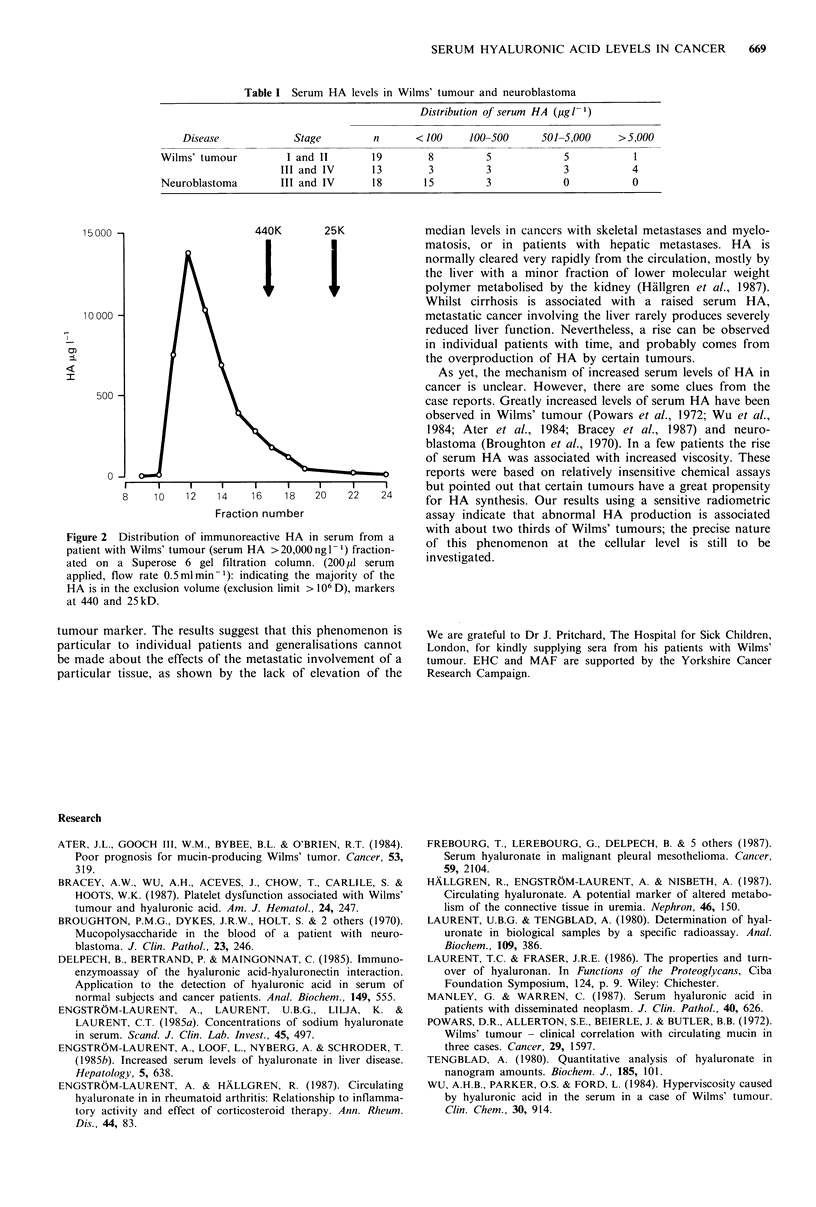# Serum hyaluronic acid levels in cancer.

**DOI:** 10.1038/bjc.1988.283

**Published:** 1988-11

**Authors:** E. H. Cooper, M. A. Forbes

**Affiliations:** Unit for Cancer Research, University of Leeds, UK.


*Br. J. Cancer* (1988), **58**, 668–669 © The Macmillan Press Ltd., 1988

SHORT COMMUNICATION

### Serum hyaluronic acid levels in cancer

E.H. Cooper & M.A. Forbes

*Unit for Cancer Research, University of Leeds, Leeds LS2 9NL, UK.*

Hyaluronic acid is an unbranched high molecular weight polysaccharide, consisting of repeating disaccharide units of glucuronate – 1, 3N acetyl glucosamine linked by 1–4 bonds.

The introduction of sensitive assays has now made it possible to detect HA in very low concentrations (Engström-Laurent *et al.*, 1985*a*; Delpech *et al.*, 1985). Tissue HA enters the circulation via the lymph and is rapidly extracted and catabolised by the liver (Laurent & Fraser, 1986), resulting in a normal serum HA level of 10–100 $\mu g l^{-1}$. The level of HA can rise in cirrhosis (Engström-Laurent *et al.*, 1985*b*) and rheumatoid arthritis (Engström-Laurent & Hällgren, 1985) and in end stage renal failure (Hällgren *et al.*, 1987).

There have been a few reports of raised serum levels of HA in advanced cancer; Delpech *et al.* (1985) made this observation when developing their enzymoimmunologic assay. Later they used this test to investigate the HA concentrations in the serum and pleural effusions in mesothelioma and observed high levels in advanced disease (mean 750 $\mu g l^{-1}$; range 29–5,833 $\mu g l^{-1}$), but the test was unsuitable for early diagnosis (Frebourg *et al.*, 1987). Manley & Warren (1987) using a nephelometric assay also observed raised serum HA levels in cancer, but their technique gave much higher concentrations for normal HA levels (mean 1.09 $mg l^{-1}$; range 0–4 $mg l^{-1}$).

The availability of a commercial radiometric (HA test 50, Pharmacia, Uppsala, Sweden) has provided an opportunity to extend these preliminary observations. This assay has been developed from the method described by Tengblad (1980) and Laurent & Tengblad (1980).

The test is based on the use of specific hyaluronic acid binding proteins, (HABP) isolated from bovine cartilage. First the hyaluronic acid from the sample is allowed to bind $^{125}$I-labelled HABP in solution for at least 60 min. The unbound $^{125}$I- HABP is then quantitated by incubating with HA covalently coupled to sepharose particles of small size and low density. The particles stay suspended during the 45 min incubation. Separation is performed by centrifugation followed by decanting. The radioactivity bound to the particles is measured. It is inversely proportional to the concentration of HA in the sample. The assay has a detection limit of $<5 \mu g l^{-1}$, and an operating range of $<5$–500 $\mu g l^{-1}$. The coefficient of variation within and between assays was 6.5 and 4.9% respectively for a sample with a mean value of 29.0 $\mu g l^{-1}$, 5.2 and 8.0% respectively for a sample with a mean value of 312 $\mu g l^{-1}$. The assay measures HA with a wide range of relative molecular weights ranging from $<10^6 - 5 \times 10^3$ kD. The manufacturer's data indicate that there is a marked age dependency for the normal values rising from a mean of 18–69 $\mu g l^{-1}$ as age rose from $<20$–$>60$ years old. We chose our controls from healthy persons between 42 and 64 years, median 54 years.

The distribution of serum HA values in the controls and 121 adult patients with metastatic cancer or large local tumours at presentation is shown in Figure 1. It is evident that within each type of cancer individual patients can show strongly elevated levels of HA, but statistical significance

**Figure 1** Distribution of serum HA levels in advanced cancer.

(Spearman rank test) for an overall increased level compared to the controls was only present in pancreatic cancer ($P=0.0097$), small cell lung cancer ($P=0.01$) and carcinoma of the prostate ($P=0.0069$). Sera from 20 patients with hepatic metastases gave a median HA of 74 $\mu g l^{-1}$ and range, 21–970 $\mu g l^{-1}$; and sera from 20 patients with metastases involving bone had a median HA of 66.5 $\mu g l^{-1}$ and range, 3–789 $\mu g l^{-1}$; both were not significantly different to normal by a non-parametric analysis.

Whilst the patients with raised HA ($>130$ $ng l^{-1}$) tended to have other tumour markers that were raised, there were other patients with normal HA levels and raised marker levels, e.g., in carcinoma of the pancreas HA *vs.* CEA ($>5$ $ng ml^{-1}$) 9:12, in colorectal 2:6, in prostate cancer HA *vs.* PSA ($>10$ $ng ml^{-1}$) 7:11 in small cell lung cancer HA *vs.* NSE ($>13$ $ng ml^{-1}$) 8:18. The induction of clinical and biochemical remission in metastatic prostatic cancer by hormone manipulation was not associated with any significant change in HA level as shown by a paired *t*-test for 9 paired values ($P=0.2865$).

The levels of serum HA are age dependent; in persons $<20$ years the 95th percentile is 37 $\mu g l^{-1}$. When sera from patients with untreated Wilms' tumour or neuroblastoma were measured it was evident that Wilms' tumour patients had a high incidence of greatly raised HA levels; in over a third the levels were $>500 \mu g l^{-1}$, and in 4 of them $>20{,}000 \mu g l^{-1}$. In neuroblastoma, by contrast, the pattern was similar to that seen in adult cancers with a median level of 25 $\mu g l^{-1}$ and range, 11–327 $\mu g l^{-1}$ (Table I). The raised levels of HA in Wilms' tumour did not appear to be stage dependent, levels $>500 \mu g l^{-1}$ occurring in some patients with all stages. The molecular weight distribution of HA in the sera of 2 patients with Wilms' tumours was analysed by high performance gel chromatography (Superose 6, Pharmacia). Both samples showed that the predominant form of HA was a high molecular weight polymer ($>10^6$ kD) and this was associated with a wide range of smaller polymers to $\sim 10^4$ kD (Figure 2).

These studies confirm that a raised serum HA may accompany malignant disease but cannot be considered as a

Correspondence: E.H. Cooper.
Received 16 April 1988; and in revised form, 15 July 1988.

**Table I** Serum HA levels in Wilms' tumour and neuroblastoma

| *Disease* | *Stage* | *n* | *Distribution of serum HA* ($\mu g\,l^{-1}$) *<100* | *100–500* | *501–5,000* | *>5,000* |
|---|---|---|---|---|---|---|
| Wilms' tumour | I and II | 19 | 8 | 5 | 5 | 1 |
| | III and IV | 13 | 3 | 3 | 3 | 4 |
| Neuroblastoma | III and IV | 18 | 15 | 3 | 0 | 0 |

**Figure 2** Distribution of immunoreactive HA in serum from a patient with Wilms' tumour (serum HA $>20,000\,ng\,l^{-1}$) fractionated on a Superose 6 gel filtration column. ($200\,\mu l$ serum applied, flow rate $0.5\,ml\,min^{-1}$): indicating the majority of the HA is in the exclusion volume (exclusion limit $>10^6$ D), markers at 440 and 25 kD.

tumour marker. The results suggest that this phenomenon is particular to individual patients and generalisations cannot be made about the effects of the metastatic involvement of a particular tissue, as shown by the lack of elevation of the median levels in cancers with skeletal metastases and myelomatosis, or in patients with hepatic metastases. HA is normally cleared very rapidly from the circulation, mostly by the liver with a minor fraction of lower molecular weight polymer metabolised by the kidney (Hällgren *et al.*, 1987). Whilst cirrhosis is associated with a raised serum HA, metastatic cancer involving the liver rarely produces severely reduced liver function. Nevertheless, a rise can be observed in individual patients with time, and probably comes from the overproduction of HA by certain tumours.

As yet, the mechanism of increased serum levels of HA in cancer is unclear. However, there are some clues from the case reports. Greatly increased levels of serum HA have been observed in Wilms' tumour (Powars *et al.*, 1972; Wu *et al.*, 1984; Ater *et al.*, 1984; Bracey *et al.*, 1987) and neuroblastoma (Broughton *et al.*, 1970). In a few patients the rise of serum HA was associated with increased viscosity. These reports were based on relatively insensitive chemical assays but pointed out that certain tumours have a great propensity for HA synthesis. Our results using a sensitive radiometric assay indicate that abnormal HA production is associated with about two thirds of Wilms' tumours; the precise nature of this phenomenon at the cellular level is still to be investigated.

We are grateful to Dr J. Pritchard, The Hospital for Sick Children, London, for kindly supplying sera from his patients with Wilms' tumour. EHC and MAF are supported by the Yorkshire Cancer Research Campaign.

**Research**

ATER, J.L., GOOCH III, W.M., BYBEE, B.L. & O'BRIEN, R.T. (1984). Poor prognosis for mucin-producing Wilms' tumor. *Cancer*, **53**, 319.

BRACEY, A.W., WU, A.H., ACEVES, J., CHOW, T., CARLILE, S. & HOOTS, W.K. (1987). Platelet dysfunction associated with Wilms' tumour and hyaluronic acid. *Am. J. Hematol.*, **24**, 247.

BROUGHTON, P.M.G., DYKES, J.R.W., HOLT, S. & 2 others (1970). Mucopolysaccharide in the blood of a patient with neuroblastoma. *J. Clin. Pathol.*, **23**, 246.

DELPECH, B., BERTRAND, P. & MAINGONNAT, C. (1985). Immunoenzymoassay of the hyaluronic acid-hyaluronectin interaction. Application to the detection of hyaluronic acid in serum of normal subjects and cancer patients. *Anal. Biochem.*, **149**, 555.

ENGSTRÖM-LAURENT, A., LAURENT, U.B.G., LILJA, K. & LAURENT, C.T. (1985*a*). Concentrations of sodium hyaluronate in serum. *Scand. J. Clin. Lab. Invest.*, **45**, 497.

ENGSTRÖM-LAURENT, A., LOOF, L., NYBERG, A. & SCHRODER, T. (1985*b*). Increased serum levels of hyaluronate in liver disease. *Hepatology*, **5**, 638.

ENGSTRÖM-LAURENT, A. & HÄLLGREN, R. (1987). Circulating hyaluronate in in rheumatoid arthritis: Relationship to inflammatory activity and effect of corticosteroid therapy. *Ann. Rheum. Dis.*, **44**, 83.

FREBOURG, T., LEREBOURG, G., DELPECH, B. & 5 others (1987). Serum hyaluronate in malignant pleural mesothelioma. *Cancer*, **59**, 2104.

HÄLLGREN, R., ENGSTRÖM-LAURENT, A. & NISBETH, A. (1987). Circulating hyaluronate. A potential marker of altered metabolism of the connective tissue in uremia. *Nephron*, **46**, 150.

LAURENT, U.B.G. & TENGBLAD, A. (1980). Determination of hyaluronate in biological samples by a specific radioassay. *Anal. Biochem.*, **109**, 386.

LAURENT, T.C. & FRASER, J.R.E. (1986). The properties and turnover of hyaluronan. In *Functions of the Proteoglycans*, Ciba Foundation Symposium, 124, p. 9. Wiley: Chichester.

MANLEY, G. & WARREN, C. (1987). Serum hyaluronic acid in patients with disseminated neoplasm. *J. Clin. Pathol.*, **40**, 626.

POWARS, D.R., ALLERTON, S.E., BEIERLE, J. & BUTLER, B.B. (1972). Wilms' tumour – clinical correlation with circulating mucin in three cases. *Cancer*, **29**, 1597.

TENGBLAD, A. (1980). Quantitative analysis of hyaluronate in nanogram amounts. *Biochem. J.*, **185**, 101.

WU, A.H.B., PARKER, O.S. & FORD, L. (1984). Hyperviscosity caused by hyaluronic acid in the serum in a case of Wilms' tumour. *Clin. Chem.*, **30**, 914.